# Geographical differences on the mortality impact of heat waves in Europe

**DOI:** 10.1186/1476-069X-9-38

**Published:** 2010-07-16

**Authors:** Jordi Sunyer

**Affiliations:** 1 Centre for Research on Environmental epidemiology. CREAL-Barcelona, Spain

## Abstract

Climate change is potentially the biggest global health threat in the 21st century. Deaths related with heat waves and spread of infectious diseases will be part of the menace though the major impact will be caused by malnutrition, diarrhea and extreme climate events. Consequently, loss of healthy life years as a result of global climate change is predicted to be 500 times greater in poor African populations than in European populations. However, the increase of more than 2°C of average temperature will result in a negative health impact in all regions, the potential benefits of a warmer temperature being negatively compensated, heat waves being one of the largest climate change threats in the developed world.

## Commentary

Climate change is potentially the biggest global health threat in the 21^st ^century [1]. Deaths related with heat waves and spread of infectious diseases will be part of the menace though the major impact will be caused by malnutrition, diarrhoea and extreme climate events [2]. Consequently, loss of healthy life years as a result of global climate change is predicted to be 500 times greater in poor African populations than in European populations [3]. However, the increase of more than 2°C of average temperature will result in a negative health impact in all regions, the potential benefits of a warmer temperature being negatively compensated, heat waves being one of the largest climate change threats in the developed world [4].

D'Ippoliti et al investigated heat waves in Europe and provide data related to several of the uncertainties of their health impact [5]. The study of D'Ippoliti et al aimed assessing for the first time the impact of heat waves in 9 European cities using the same criteria to define heat wave and the same methodology to asses the mortality association. The study refers to the summer months of the years 1990-2004. Heat wave was defined based on the city 90^th ^percentile of the monthly maximum and minimum apparent temperature.

The major finding refers to the great heterogeneity in the effect of heat waves on diary mortality. The strongest effect was seen in the Mediterranean cities (Athens, Barcelona, Milan, Rome and Valencia) than in the North-Continental (Budapest, London, Munich and Paris) after adjusting for age, sex and cause of death. The authors suggested higher temperatures could explain higher effects in the South, but this could not account for the large variability within Mediterranean cities (i.e., the effect is lower in Valencia (increase of 8.5% of total mortality) than in Milan (33.6%), Rome (26.8%) or Barcelona (15.6%)). Similarly, among the non-Mediterranean cities the heterogeneity is also large, from Munich (7.6%) to Budapest (21.1%). Furthermore, intensity of the heat waves (based on temperatures below or above the 95^Th ^percentile) only explains a small part of the effect of the heat waves in this study.

A complementary explanation could be that the type of heat waves defined by duration or seasonality explains the geographical heterogeneity. The authors found that long duration (defined by waves longer than the median) play a bigger role in the morality effect than intensity while seasonality of the heat wave did not produce homogeneous result in the association with mortality. However duration, intensity or timing, or air pollution concentrations did not explain the heterogeneity between cities in the mortality effects of heat waves. A problem of present study could be residual confounding since type of heat waves (i.e., intensity, duration and seasonality) was categorized only in two and three categories, respectively.

The 2003 was an exceptional year in most of the cities but no in all (such as Athens, Budapest, London) which number of heat days was within the range observed in the period 1990-2002. The effect of the heat waves in that year was larger than in the rest of years in all cities except in Athens, Budapest and Munich. The relative increase was 20 times higher in Paris in comparison with the heat wave effects of the previous years and five times higher in London and Barcelona. The authors speculated that adaptation to the heat could explain a lower relative impact in Milan, Rome or Valencia than in Paris or London. However this explanation did no account for the large effect in Barcelona. There are data from Sweden [6] suggesting that the amount of frailty population at risk at the time of the heat wave could explain the heterogeneity in the impact. One of the explanations for the high mortality occurring in summer of 2003 was the lack of influenza the previous winter. The lack of data on harvesting did not allow contrasting this potential effect in the D'Ippolity study.

In all areas, mortality due to heat waves increased by age, in general whatever the cause and the gender. Several biological mechanisms have been postulated for susceptible populations to heat-related mortality, particularly the elderly [7]. The main underlying mechanism is that stress on the cardiovascular and respiratory systems increases during periods of high ambient temperature, especially among elderly persons with limited ability to thermoregulate body temperatures and elevated sweating thresholds. Vulnerability was poorly studied by D'Ipoltiti et al. They have studied few individual characteristics (age, sex, cause of death) which did not explain the geographical variability of the heat wave effect.

Contextual characteristics could explain the geographical differences both among the usual heat waves and in the exceptional heat wave occurring in 2003. Variables related with the health system, the housing conditions, the elderly care, the type of urbanisation, construction material, insulation, air conditioning, and the plans of action are among the variables not studied by D'Ippolity et al, which impact deserves further research. Their knowledge is important in order to implement the protection plans required to face the increase in heat waves forecast for the forthcoming decades.

In fact, after the heat wave of 2003 in Europe, intervention programs have been adopted in many countries which have been proved effective in the reduction of deaths in the heat wave of 2006 in France [8], as well as in previous evaluation studies of preparedness head wave programs in Milwakke (USA) [9] and Shangai (China) [10]. Given that changes in public health preparedness and response have contributed to reduce mortality and morbidity, a lesson that D'Ippoliti et al. provides for decision-making is that all regions have to be prepared for preventing heat wave health effects. Their 2003 data showed mortality increases markedly in Paris and London, while the usual summers showed that Mediterranean cities had the largest increase of mortality related to heat waves.

## Competing interests

The author declares that they have no competing interests.
